# Radiotherapy With Hydrogen Peroxide-Soaked Gauze for Unresectable Breast Cancer

**DOI:** 10.7759/cureus.19167

**Published:** 2021-10-31

**Authors:** Takahiro Oike, Kento Tomizawa, Akiko Adachi, Masahiro Wada, Tatsuya Ohno

**Affiliations:** 1 Heavy Ion Medical Center, Gunma University, Maebashi, JPN; 2 Department of Radiation Oncology, Sano Kousei General Hospital, Sano, JPN; 3 Department of Radiation Oncology, Gunma University Graduate School of Medicine, Maebashi, JPN; 4 Department of Breast Surgery, Sano Kousei General Hospital, Sano, JPN

**Keywords:** bleeding, hydrogen peroxide, palliation, breast cancer, radiotherapy

## Abstract

A hydrogen peroxide (H_2_O_2_)-soaked gauze has been used in combination with radiotherapy in anticipation of sensitizing tumors exposed to the skin surface. Although used empirically in the clinic, the method is rarely reported in the literature, making its efficacy and tolerability unclear. Here, we report a case of primary metastatic breast cancer whose primary tumor was treated with palliative radiotherapy using an H_2_O_2_-soaked gauze. The primary tumor in the right breast regrew after treatment with palbociclib plus letrozole followed by fulvestrant and denosumab. The tumor was exposed to the skin surface, causing exudation, bleeding, pain, and difficulty in raising the right upper limb. Radiotherapy (51 Gy in 17 fractions) using the H_2_O_2_-soaked gauze resolved the patient's symptoms and the tumor showed macroscopic complete remission at three months post-treatment. This case indicates that radiotherapy with an H_2_O_2_-soaked gauze is an effective and tolerable palliative treatment for superficially exposed tumors. This non-invasive and inexpensive method of radiosensitization warrants validation and optimization in a prospective setting.

## Introduction

Radiotherapy is used to treat tumors in frail or elderly individuals who are not suitable for or refuse surgery. However, it is often difficult for radiotherapy alone to eradicate tumors or even to relieve tumor-related symptoms such as bleeding and pain. Thus, there is an unmet clinical need for novel methods of radiosensitization.

Preclinical evidence suggests that hydrogen peroxide (H_2_O_2_) sensitizes cancer cells to X-rays [1,2]. A study in a mouse tumor model showed that intratumoral injection of 0.5% H_2_O_2_ (in 0.83% sodium hyaluronate gel) combined with X-rays resulted in greater growth suppression than X-rays alone [3]. A recent phase one clinical trial analyzing 12 inoperable patients with breast cancer demonstrated that intratumoral injection of H_2_O_2_ plus radiotherapy was tolerable [4]. Based on these data, a phase two clinical trial is underway.

On the other hand, an H_2_O_2_-soaked gauze has been used in combination with radiotherapy in anticipation of sensitizing the tumors exposed to the skin surface. Although this method is used empirically in the clinic, the use of an H_2_O_2_-soaked gauze is rarely reported in the literature (in contrast to intratumoral injection of H_2_O_2_); thus, the efficacy and tolerability of the bolus method are unclear. Here, we report a case of primary metastatic breast cancer whose primary tumor was treated with palliative radiotherapy plus an H_2_O_2_-soaked gauze.

## Case presentation

A 77-year-old woman was referred to our radiation oncology unit for palliative treatment of a recurrent primary tumor in the right breast. The tumor caused exudation, bleeding, pain, and difficulty in raising the right upper limb, thereby reducing the patient's quality of life (QOL).

**Figure 1:**
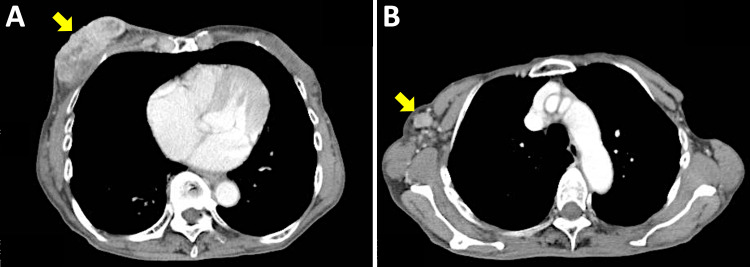
Contrast-enhanced computed tomography image obtained at the time of referral to the radiation oncology unit. (A) The primary tumor in the right breast (70 x 24 mm). (B) Metastasis to a right axial lymph node (15 x 11 mm). Arrows indicate the tumors.

Nineteen months before the referral, the patient visited the surgical oncology unit and was newly diagnosed with stage IV (T2N0M1) breast cancer with lung metastases. The primary tumor occupied the lateral half of the right breast. The tumor was 47 mm in diameter, as assessed by computed tomography (CT), and was estrogen and progesterone receptor-positive, and human epidermal growth factor receptor-2 (HER2)-negative. The patient developed bone metastasis after four courses of palbociclib plus letrozole as first-line treatment. After 13 courses of fulvestrant as second-line treatment combined with monthly denosumab, the stable primary tumor became progressive, with axially lymph node metastasis (Figure 1). The primary tumor was considered inoperable, resulting in a referral to the radiation oncology unit.

**Figure 2:**
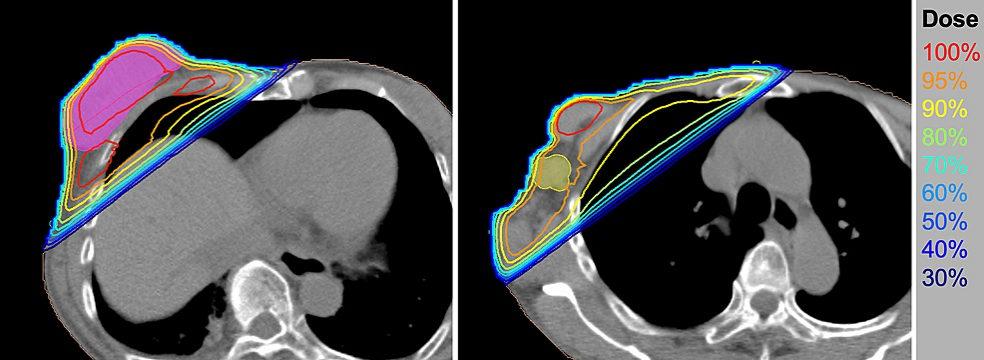
Treatment plan for radiotherapy. (A) A plane showing the primary tumor (solid magenta). (B) A plane showing the axial lymph node metastasis (solid yellow). Isodose lines are shown on computed tomography images.

**Figure 3:**
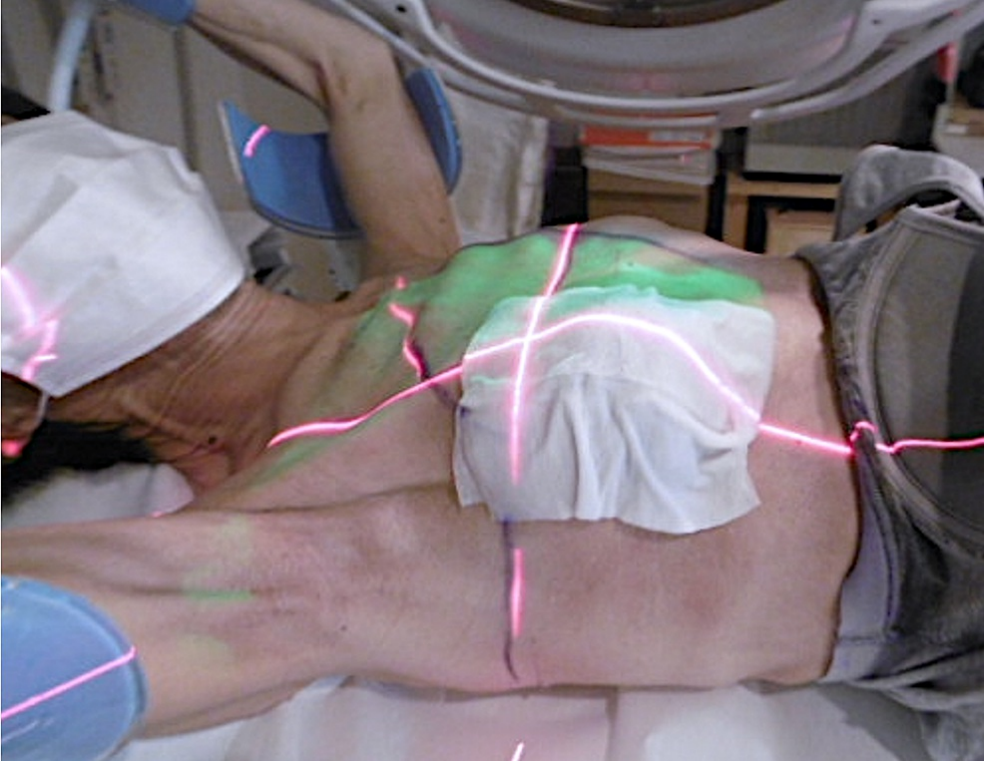
Application of the hydrogen peroxide-soaked gauze during the radiotherapy session.

Employing standard tangentially opposed irradiation techniques, we performed conventional three-dimensional conformal radiotherapy with 6-MV photons [4]. The gross tumor, metastatic node, and tumor bed received 51 Gy delivered in 17 fractions (five fractions per week) (Figure 2). At each radiotherapy session, an H_2_O_2_-soaked gauze (approximately 5 mm thick) was freshly prepared and placed on the right breast to cover the tumor (Figure 3). Practically, when the patient entered the treatment room, a nurse poured oxydol (i.e., 2.5-­3.5 w/v% H_2_O_2_; KENEI Pharmaceutical Co. Ltd., Osaka, Japan) in a plastic bag and a gauze was soaked in the bag. As the treatment progressed, the tumor showed a fair amount of shrinkage. Exudation and bleeding from the tumor, as well as the pain and difficulty in raising the right upper limb, resolved upon completion of the treatment. The patient experienced grade one radiation dermatitis (assessed by the Common Terminology Criteria for Adverse Events 4.0) but did not experience pain from the application of the H_2_O_2_ gauze. The dermatitis was resolved post-radiotherapy without topical medication.

**Figure 4:**
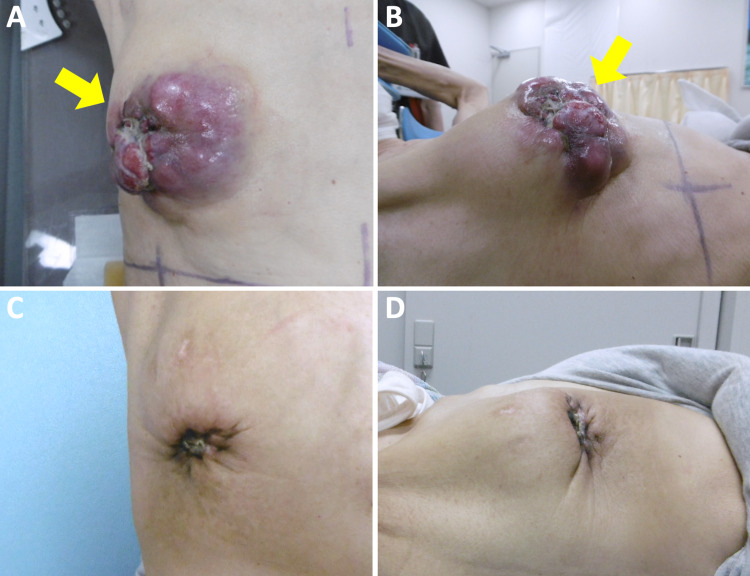
Pictures of the patient's right breast. (A, B) Radiotherapy day one. Arrows indicate the primary tumor. (C, D) Three months post-completion of radiotherapy.

**Figure 5:**
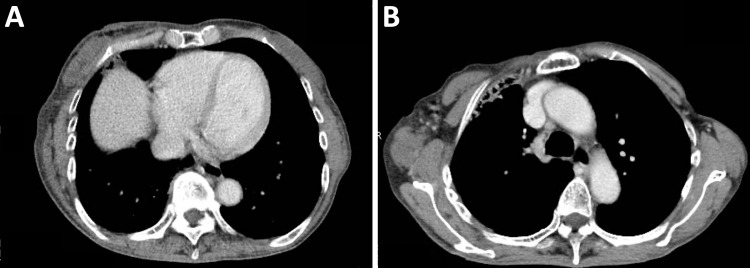
Contrast-enhanced computed tomography image obtained at four months post-treatment. (A) Primary tumor site. (B) Metastatic lymph node site.

**Figure 6:**
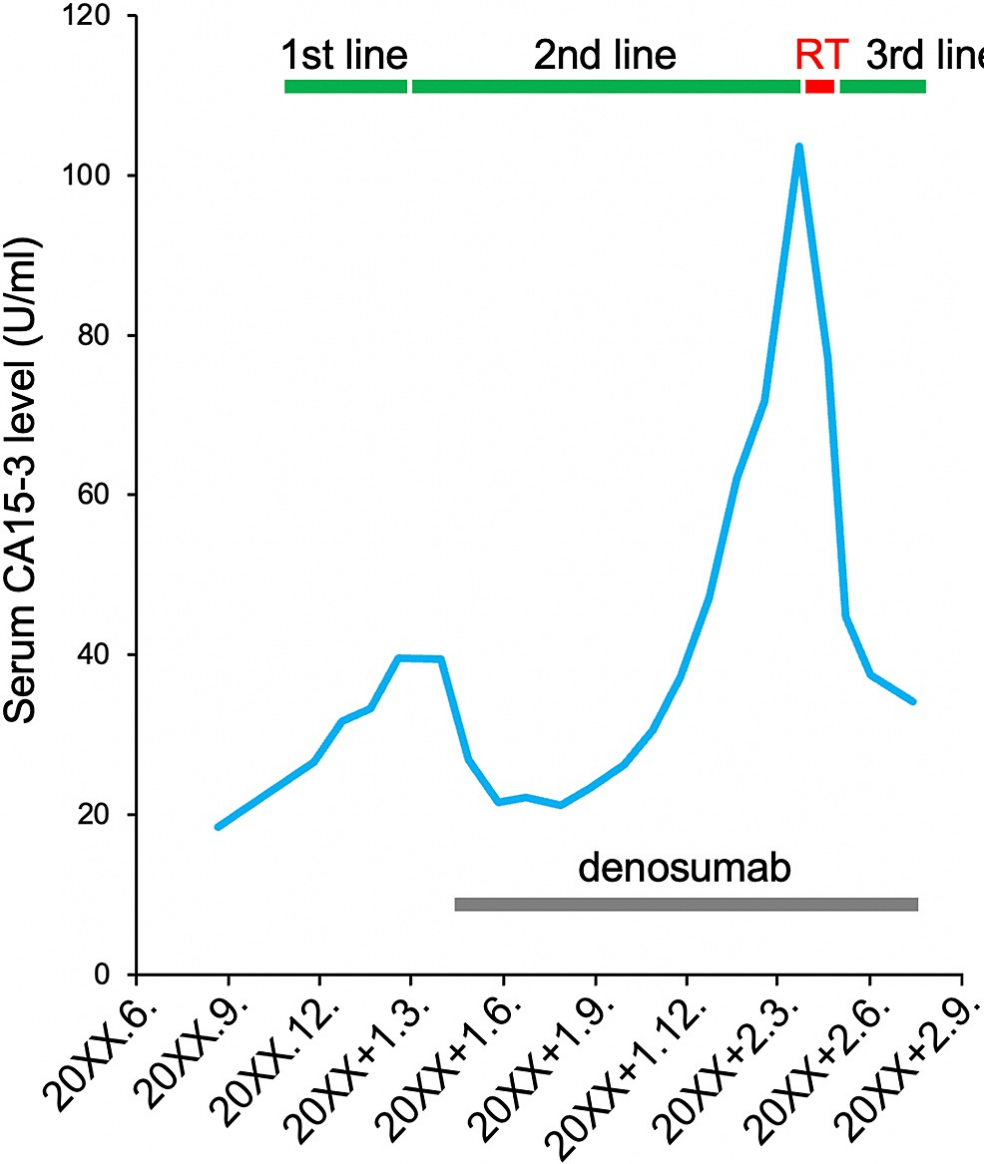
Serum CA15-3 kinetics. 1st line, palbociclib plus letrozole; 2nd line, fulvestrant; 3rd line, tegafur; RT, radiotherapy with the hydrogen peroxide bolus; CA15-3, cancer antigen 15-3.

Upon completion of radiotherapy, tegafur was started as a third-line treatment. At three months post-completion of radiotherapy, the tumor showed macroscopic complete remission (Figure 4). The irradiated tumor was almost unidentifiable in CT images obtained at four months post-completion of radiotherapy (Figure 5). Serum cancer antigen 15-3 (CA15-3), a marker reflecting disease activity, also showed a substantial decrease after radiotherapy (Figure 6). The patient has resumed her daily life without symptoms and with peace of mind, although the progressive disease is present in other organs.

## Discussion

Breast cancer exposed to the skin surface threatens QOL severely by causing various symptoms including bleeding and pain. Such cases are often medically inoperable. In addition, clinical evidence suggests that, at least for breast cancer, approximately half of the tumor volume is hypoxic; hypoxia-enriched tumors are radioresistant [5]. Therefore, radiotherapy alone may be insufficient to resolve the symptoms, especially in the case of bulky tumors. In our case, radiotherapy with an H_2_O_2_-soaked gauze led to temporal complete remission of a bulky breast tumor exposed to the skin surface, with minimal toxicity; these findings suggest the potential efficacy and tolerability of this treatment for palliation.

The radiosensitization strategy using an H_2_O_2_-soaked gauze was first described by Ogawa and colleagues in 2008 [6]. In that paper, the authors reported five cases of locally advanced and/or recurrent tumors exposed to the skin surface, which were treated with electrons plus an H_2_O_2_-soaked gauze (Table 1). A few other cases treated with the same strategy have been reported in a Japanese book (Table 1). In the previously reported cases, the equivalent dose in 2 Gy per fraction with an α/β ratio of 10 (EQD2_α/β10_) ranged from 54 Gy to 64 Gy. All cases showed a partial or complete response. A total of 85% (6/7) of the cases treated using EQD2_α/β10_ equal to or less than 56 Gy experienced grade one radiation dermatitis, whereas one patient with penile cancer treated using 64 Gy in 32 fractions experienced grade three radiation dermatitis. In our case, the EQD2_α/β10_ was 55.2 Gy and the treatment resulted in temporal complete tumor remission, with grade one radiation dermatitis. These data indicate that the use of an H_2_O_2_-soaked gauze is tolerable when administered alongside an EQD2_α/β10_ of approximately <60 Gy. Nevertheless, solid evidence of tolerable and optimal dose fractionation for this treatment is still lacking, thereby warranting further research in a prospective setting.

**Table 1:** Summary of previously reported cases of superficial tumors treated with radiotherapy using a hydrogen peroxide bolus. fr., fraction; EQD2_α/β10_, the equivalent dose in 2 Gy per fraction with α/β of 10; M, months; Refs, references; NA, not assessed; PR, partial response; CR, complete response. Adverse effect was assessed by the Common Terminology Criteria for Adverse Events 4.0, where G indicates grade. *Japanese book (ISBN: 978-4-86705-804-6).

Cancer type	n	Radiation	Dose/fr.	EQD2_α/β10_	Follow-up (M)	Outcome	Adverse effect	Refs
Breast cancer	1	Electrons	48 Gy/12 fr.	56 Gy	3	PR	Dermatitis (G1)	[6]
Melanoma	1	Electrons	48 Gy/12 fr.	56 Gy	12	CR	Dermatitis (G1)	[6]
Malignant fibrous histiocytoma	1	Electrons	48 Gy/12 fr.	56 Gy	3	PR	Dermatitis (G1)	[6]
Skin squamous cell carcinoma	1	Electrons	48 Gy/12 fr.	56 Gy	1	PR	Dermatitis (G1)	[6]
Extramammary Paget's disease	1	Electrons	48 Gy/12 fr.	56 Gy	12	CR	Dermatitis (G1)	[6]
Angiosarcoma	2	Electrons	52.5 Gy/21 fr.	54.7 Gy	NA	NA	Dermatitis (G2, 50%)	*
Malignant trichilemmoma	1	X-rays	54 Gy/27 fr.	54 Gy	3	CR	NA	*
Penile cancer	1	X-rays	64 Gy/32 fr.	64 Gy	NA	CR	Dermatitis (G3)	*
Breast cancer	1	X-rays	51 Gy/17 fr.	55.2 Gy	4	CR	Dermatitis (G1)	Present case

Although the detailed mechanisms underlying the radiosensitizing effect of H_2_O_2_ are currently being investigated in the laboratory setting, the putative mechanism is as follows: first, two molecules of H_2_O_2_ break down to yield one molecule of oxygen and two molecules of water. This process can contribute to the reoxygenation of the radioresistant hypoxic tumor microenvironment. Second, H_2_O_2_ in combination with ionizing radiation induces intracellular production of reactive oxygen species (ROS). This can increase the induction of DNA double-strand breaks (i.e., the indirect effect). In addition, it is possible that ROS activate inflammatory signals that may upregulate antitumor immune responses [7]. Third, H_2_O_2_ influences a number of radioresistance-associated signaling pathways, including extracellular signal-regulated kinase (ERK), mitogen-activated protein kinase (MAPK), and nuclear factor kappa B (NFkB) [8-10]. Further research is needed to fully elucidate the underlying mechanisms and the optimal method of radiosensitization by H_2_O_2_.

As the limitation of this case report, first, there is a possibility that the H_2_O_2_-soaked gauze just functioned to improve dose distribution as same as water-soaked gauze. In fact, a water-density 5-mm thick structure improved the dose distribution near the skin in this case (data not shown). To address this issue, a randomized study, that employs water-soaked gauze as control, is mandatory. Second, we used a commercially available oxydol (i.e., 2.5­3.5 w/v% H_2_O_2_) according to the original report [6]; nevertheless, there is a possibility that the concentration of H_2_O_2_ can be further optimized so that it achieves maximal radiosensitizing effect with tolerable adverse effect, warranting further research. Third, in our case, we cannot exclude the possibility that tegafur contributed to the fair tumor response post-radiotherapy. Lastly, the possibility that the present case was an exceptional responder to ionizing radiations cannot be excluded, warranting validation by a prospective study.

## Conclusions

An H_2_O_2_-soaked gauze has been used in combination with radiotherapy in anticipation of sensitizing the tumor exposed to the skin surface. Although this method is used empirically in the clinic, the use of an H_2_O_2_-soaked gauze is rarely reported in the literature (in contrast to intratumoral injection of H_2_O_2_); thus, the efficacy and tolerability of the gauze method are unclear. Here, we report a case of primary metastatic breast cancer whose primary tumor was treated with palliative radiotherapy plus an H_2_O_2_-soaked gauze. Exudation, bleeding, pain, and difficulty in raising the right upper limb were resolved and the tumor showed macroscopic complete remission at three months post-treatment. This case indicates that radiotherapy with an H_2_O_2_-soaked gauze is both effective and tolerable as a palliative treatment for superficially exposed tumors.
